# Computational modelling distinguishes diverse contributors to aneurysmal progression in the Marfan aorta

**DOI:** 10.1098/rspa.2023.0116

**Published:** 2023-08-16

**Authors:** David S. Li, Cristina Cavinato, Marcos Latorre, Jay D. Humphrey

**Affiliations:** 1 Department of Biomedical Engineering, Yale University, New Haven, CT, USA; 2 LMGC, Univ. Montpellier, CNRS, Montpellier, France; 3 Centre for Research and Innovation in Bioengineering, Universitat Politècnica de València, València, Spain; 4 Vascular Biology and Therapeutics Program, Yale School of Medicine, New Haven, CT, USA

**Keywords:** biomedical engineering, mathematical modelling, computer modelling and simulation, elastic fibre fragmentation, mechanosensing, collagen remodelling, matrix turnover

## Abstract

Thoracic aortic aneurysms are characterized by a progressive loss of biomechanical functionality resulting from degenerative changes in wall composition, microstructure, and mechanical properties. Among the many causes of these lesions, Marfan syndrome is the most common heritable condition, resulting from mutations to the gene that codes the elastin-associated glycoprotein fibrillin-1. Key histopathological features of the aneurysmal Marfan aorta include extensive degradation of elastic fibres, significant remodelling of fibrillar collagens and compromised smooth muscle cell function. Computational growth and remodelling models have confirmed the importance of compromised elastic fibre integrity in aneurysmal dilatation, but we show here that this contributor alone is not sufficient to describe biomechanical data collected from the two most common mouse models of Marfan syndrome. Rather, our simulations suggest that compromised mechanosensing and mechanoregulation of extracellular matrix by mural cells also play central roles in the natural history. Determination of optimal disease-contributing parameters further suggests a rapid reduction in cellular mechanosensing and mechanoregulation relative to diminished elastic fibre integrity, highlighting the importance of inter- and intra-lamellar elastin in the Marfan aorta. Aneurysmal dilatation in Marfan syndrome thus results from multiple contributors to progressive degeneration of the aortic wall, and computational mechanobiological models can help disentangle these contributions.

## 1. Introduction

Marfan syndrome is a connective tissue disorder that affects approximately 1 in 5000 live births. It was discovered in 1991 that this disorder results from mutations to the gene (*FBN1*) that codes the elastin-associated glycoprotein fibrillin-1 [1–3]. Soon thereafter, mouse models revealed that fibrillin-1 mainly affects elastic fibre homeostasis, not elastogenesis [4]. That is, normal fibrillin-1 contributes to the long-term biological stability of elastic fibres, thus facilitating their previously measured remarkable half-life, on the order of decades [5]. Compromised or reduced fibrillin-1 can thus accelerate a progressive loss of elastic fibres and their vital contributions to tissue function. Although cardiovascular, musculoskeletal and ocular tissues are affected in Marfan syndrome, it is dissection and rupture of the aortic root and/or ascending aorta, often secondary to aneurysmal dilatation, that is the primary cause of premature death in these patients [6,7]. There is, therefore, a pressing need for a better understanding of the aetiology, histopathology, and especially biomechanics of the Marfan aorta.

The two most common animal models of Marfan syndrome are *Fbn1^mgR/mgR^* mice, which are hypomorphic (approx. 20%) for normal fibrillin-1 [4], and *Fbn1*^*C1041G/*+^ mice, which model a heterozygous mutation in Marfan patients [8]. Much has been learned from these two mouse models via quantification of changes in the histology and biomechanics of the thoracic aorta (e.g. [9–14]). Data from these mice have also motivated computational modelling, including our own recent study of possible mechanobiological contributors to thoracic aortic aneurysm (TAA) progression [15]. In particular, motivated further by recognition that many TAAs result in part from dysfunctional cellular mechanosensing and mechanoregulation [16–19]—that is, a reduced ability of vascular cells to assess (sense) and assemble (regulate) the local extracellular matrix via integrins and actomyosin activity [20–22]—we previously examined computationally whether locally compromised elastic fibre integrity, collagen cross-linking, smooth muscle contractility, or mechanosensing and mechanoregulation of matrix can give rise to aortic dilatation, and whether hypertension and vascular ageing can be contributing factors. The resulting quantitative predictions were highly supportive of recent hypotheses regarding these many factors, but in the absence of appropriate data, the simulations remained hypothetical.

In this paper, we use recently published histological and biaxial biomechanical data from *Fbn1*^*C1041G/*+^ and *Fbn1^mgR/mgR^* mice [13] (denoted in figures and tables as C1041G/+ and mgR/mgR, respectively) to parametrize our computational model of aneurysm progression. Although our current simulations confirmed that multiple factors can give rise to aortic dilatation, capturing the actual data required that multiple factors coexist. That is, modelling diminished elastic fibre integrity alone did not recapitulate the observed changes in vessel geometry and mechanical properties; however, when considering compromised elastic fibres in combination with dysfunctional mechanosensing and mechanoregulation, the model successfully reproduced the experimental findings. We submit, therefore, that modelling of contributing mechanobiological factors in addition to structural defects can offer deeper insight into the mechanisms driving aneurysmal dilatation within the Marfan ascending aorta.

## 2. Methods

### (a) Constitutive relations

TAAs are progressive lesions, enlarging over weeks to months in mice and perhaps over months to years in humans. There is a need, therefore, for a computational framework that can simulate evolving changes in arterial geometry, composition, and material properties; we used a so-called constrained mixture framework for soft tissue growth (changes in mass) and remodelling (changes in structure). Introduced 20 years ago [23], this general framework enforces quasiequilibrium at the tissue level (divσ=0, where $\sigma =2F(\partial W/\partial C)F^{T}/det\;F$ is the Cauchy stress tensor, **F** the deformation gradient tensor, $C=F^{T}F$ the right Cauchy–Green tensor, and *W* a scalar stored energy function) while exploiting the concept of a constrained mixture, namely, that α=1,2,…,N structurally significant constituents are allowed to possess individual natural configurations and to manifest individual material properties and rates of turnover but are constrained to move with the mixture as a whole. It is also assumed that the individual constituents satisfy individual mass balance relations, while the overall stored energy function can be computed according to a rule-of-mixtures simply as the sum of the constituent-specific stored energies: $W=\sum_{\alpha =1}^{N}W^{\alpha }$. This approach has proven useful in diverse applications in soft tissue mechanics and mechanobiology in general [24] and in vascular studies in particular [25].

Briefly, it can be shown that a useful form for the constituent-specific stored energy functions is

(2.1) $$\rho (s)W^{\alpha }(s)=\rho^{\alpha }(0)Q^{\alpha }(s){\overset{ˆ}{W}}^{\alpha }\left(F_{n(0)}^{\alpha }(s)\right)+\int_{0}^{s}m^{\alpha }(\tau )q^{\alpha }(s,\tau ){\overset{ˆ}{W}}^{\alpha }\left(F_{n(\tau )}^{\alpha }(s)\right)d\tau ,$$

where ρ(s) is the mixture mass density at any growth and remodelling (G&R) time *s*, and $\rho^{\alpha }(0)$ are constituent-specific apparent mass densities at an initial time in maturity prior to any perturbation from normal. As can be seen, one must prescribe three classes of constituent-specific constitutive relations: true rates of mass density production ($m^{\alpha }(\tau )>0$), survival (removal) functions ($q^{\alpha }(s,\tau )\in [0,1]$, where $Q^{\alpha }(s)$ can be taken as $q^{\alpha }(s,0)$, where τ∈[0,s] is an intermediate time at which new constituents are produced and deposited within extant matrix), and mass-averaged stored energy functions (${\overset{ˆ}{W}}^{\alpha }(s)>0$).

Extensive studies in vascular mechanics and mechanobiology have suggested utility of the following stress-mediated relations for mass density production and removal [25]

(2.2) $$\left.\begin{array}{l} m^{\alpha }\left(\tau \right)=m_{o}^{\alpha }\left(1+K_{\sigma }^{\alpha }\Delta \sigma \left(\tau \right)-K_{\tau_{w}}^{\alpha }\Delta \tau_{w}\left(\tau \right)\right)\\ q^{\alpha }\left(s,\tau \right)=\exp\left(-\int_{\tau }^{s}k_{o}^{\alpha }\left(1+\omega \Delta \sigma^{2}\left(t\right)\right)dt\right), \end{array}\right\}$$

and

where $m_{o}^{\alpha }$ and $k_{o}^{\alpha }$ are basal values of production and removal, respectively. Moreover, Δσ and $\Delta \tau_{w}$ represent normalized deviations in scalar metrics of Cauchy stress from original homeostatic targets (denoted *o*, with $\sigma_{o}$ for intramural and $\tau_{wo}$ for wall shear stress), where it has proven useful to let

(2.3) $$\Delta \sigma =\left(\frac{(1-\delta )\sigma -\sigma_{o}}{\sigma_{o}}\right)\;\text{and}\;\Delta \tau_{w}=\left(\frac{(1-\xi )\tau_{w}-\tau_{wo}}{\tau_{wo}}\right),$$

with δ∈[0,1] and ξ∈[0,1] being mechanosensing parameters, meaning that they capture the ability of a vascular cell to assess its local mechanical environment via integrins and actomyosin activity; compromised mechanosensing is given by values (0,1]. It has also proven useful to calculate the (scalar) intramural stress σ as one-third the trace of the Cauchy stress, hence capturing circumferential and axial effects. Importantly, the gain-type parameters $K_{\sigma }^{\alpha }$ and $K_{\tau_{w}}^{\alpha }$ and similarly ω model the strength of the stress-mediated response (equation (2.2)). Lack of a mechano-response is given in the limit as these gain-type parameters tend to zero, which we have shown renders simulated vessels unable to adapt to any sustained changes in blood pressure and flow, thus reinforcing the importance of mechanical homeostasis in arteries [25].

It can be shown [25] that the constituent-specific deformation gradients $F_{n(\tau )}^{\alpha }(s)$, which depend on the individual natural configurations n(τ) (also constituent-specific), can be determined from deformation gradients experienced at the mixture level, F(τ)∀τ∈[0,s], plus a so-called deposition stretch tensor $G^{\alpha }(\tau )$, namely $F_{n(\tau )}^{\alpha }(s)=F(s)F^{-1}(\tau )G^{\alpha }(\tau )$. This deposition tensor accounts for the observation that vascular cells tend not to simply secrete new extracellular matrix into the extracellular space, but rather that they tend to work on and pre-stress newly deposited matrix through integrins via actomyosin activity [20,21]. That is, differing values of $G^{\alpha }(\tau )$ can capture one aspect of cellular mechanoregulation, with reorientation of fibre angles representing another aspect.

Finally, we must prescribe constitutive relations for the material behaviour. It has proven useful to consider two primary contributors within the aortic wall, elastin-dominated amorphous matrix (α=e) and collagen-dominated (α=c) anisotropic contributions, to which one can add circumferentially oriented smooth muscle (α=m). Specifically, we use neo-Hookean and Fung type exponential relations to describe the two dominant behaviours, respectively. That is,

(2.4) $${\overset{ˆ}{W}}^{e}=c^{e}\left(I_{1}^{e}-3\right)\;\text{and}\;{\overset{ˆ}{W}}^{m,c}=\frac{c_{1}^{m,c}}{4c_{2}^{m,c}}\left(exp\left(c_{2}^{m,c}{\left(I_{4}^{m,c}-1\right)}^{2}\right)-1\right)$$

where $\left\{c^{e},c_{1}^{m,c},c_{2}^{m,c}\right\}$ are material parameters, and $\left\{I_{1}^{e},I_{4}^{m,c}\right\}$ are coordinate invariant measures of the constituent-specific finite deformation. Note that reducing the value of $c^{e}$ can model diminished elastic fibre integrity. We model the contributions of collagen-dominated matrix with four families of fibres, oriented axially (z), circumferentially (θ), and symmetric diagonally (d) at a (potentially evolving) deposition angle $\alpha_{0}$. Conceptually, then, the total stored energy is computed using the mass fractions of each constituent $\phi^{\alpha }$, such that $W^{\alpha }=\phi^{\alpha }{\overset{ˆ}{W}}^{\alpha }$.

Given that these relations are motivated by the concept of mechanical homeostasis—namely, that certain mechanical quantities tend to be regulated to remain near target values, often called set-points—note that homeostasis requires a balanced turnover of matrix within an unchanging mechanical state and that cells deposit new matrix with a preferred pre-stress [26], that is, $m_{o}^{\alpha }=\rho_{o}^{\alpha }k_{o}^{\alpha }$, where $\rho_{o}^{\alpha }=\rho^{\alpha }(0)$, and $F_{n(\tau )}^{\alpha }(s)=F(s)F^{-1}(\tau )G^{\alpha }(\tau )\to G_{o}^{\alpha }$. We have found that deposition stretches of smooth muscle and collagen can be well represented using scalar quantities $G_{o}^{m,c}$ [27]. Importantly, reduced values of $G^{c}$ from its baseline can model compromised mechanoregulation of collagen.

### (b) Motivation

Herein we focus on the ascending thoracic aorta in Marfan syndrome given the availability of critical histological and biomechanical data in mice. Studying the *Fbn1^mgR/mgR^* mouse model, we previously found that elastic energy storage decreases and circumferential material stiffness increases with dilatation of the Marfan aorta [11]. More recent data confirmed these findings while showing similar but less dramatic trends in age-matched *Fbn1*^*C1041G/*+^ mice [13]. Importantly, in this latter study (summarized in electronic supplementary material, appendix A), we quantified three-dimensional elastic fibre damage in terms of an ‘elastin porosity’ determined from two-photon fluorescence imaging and found that increasing luminal diameter, decreasing elastic energy storage, and especially increasing circumferential material stiffness all correlate strongly with increasing elastin porosity, which increased significantly from wild-type to *Fbn1*^*C1041G/*+^ to *Fbn1^mgR/mgR^* aortas. Most elastic fibres constitute the elastic lamellar structure of the aorta [28], yet a significant fraction exists as inter- and intra-lamellar constituents that appear to have multiple functions [29], including contributing to mechanosensing by the smooth muscle cells [30,31]. Indeed, whereas the elastic lamellar structures appear mature by about postnatal day P21 in mice [5], the inter- and intra-lamellar fibres and fibrils tend to emerge from approximately P14 to maturity in rats [32], during which time intramural stress increases from low postnatal to higher mature values [33], suggesting that these fibres and fibrils arise to facilitate sensing of the increasing stress. In contrast to cross-linked elastic fibres, which have a long half-life (on the order of decades [5]), fibrillar collagens and glycosaminoglycans appear to have much shorter half-lives, with normal values on the order of months that can decrease to values on the order of days under conditions of increased mechanical stress [34]. Hence, rates of turnover and mechanoregulation of newly deposited matrix become critical parameters [35,36]. Furthermore, increasing evidence for altered integrin signalling in Marfan syndrome suggests compromised actomyosin activity in vascular cells may play a major role in disease progression [37–39].

Motivated thus, in this paper we model progressive changes in aortic structure and function of the Marfan aorta in *Fbn1*^*C1041G/*+^ and *Fbn1^mgR/mgR^* mice. (Note that, given the biaxial experimental set-up designed to minimize smooth muscle contraction and the fact that the Marfan aorta associates with dysfunctional contractility, we aimed to capture the passive properties only.) We assess parametrically the individual and combined effects of multiple contributors to aortic dilatation, including compromised elastic fibre integrity and dysfunctional mechanosensing and mechanoregulation of matrix, with the aim to capture the geometric and mechanical ranges encompassed by both experimental groups within a single mechanobiologically equilibrated, rule-of-mixtures G&R model. Table 1 shows baseline values of parameters that are essential to G&R simulations, noting that the *Fbn1* mutations are germline, not induced conditionally. That is, baseline values represent a mild form of the Marfan aorta, prior to dilatations that would be considered aneurysmal. Constituent-specific material parameters were estimated from experimental data using nonlinear least-squares regression as described previously [40] (additional details are listed in electronic supplementary material, appendix B).

### (c) Mechanobiological contributors to aortic aneurysm

We modelled aneurysm development by prescribing mechanical or mechanobiological insults (i.e. defects in vessel structure and function that predispose to aneurysm), denoted generically by ϑ, to a vessel at an initial *in vivo* state. Let ε∈[0,1] represent elastin porosity, defined as the ratio of the volume of voids to the total volume occupied by elastic fibres detected in two-photon imaging. Considering the roughly sixfold increase in elastin porosity observed over the experimental data range [13], aneurysm contributors were initially hypothesized to consist primarily of compromised elastic fibre integrity ($\vartheta_{c^{e}}$), corresponding to a reduction in the baseline value of the elastin modulus $c^{e}$ (equation (2.4)). Note that, for a mixture, the net elastic modulus is given by the product of the mass fraction and the true elastic modulus. Hence, our model captures the net loss of integrity, which can result from either loss of material (e.g. increased porosity) or loss of integrity of that material, or both. Without details on the stress-strain behaviour of individual fibres or laminae in Marfan syndrome, using a reduction in the net modulus is preferred.

For the purposes of the simulations, we let elastic fibre integrity reduction progress proportionally to increases in elastin porosity, namely

(2.5) $$\vartheta_{c^{e}}=\bar{\varepsilon }\vartheta_{c^{e}}^{max},$$

where $\bar{\varepsilon }=\left(\varepsilon -\varepsilon_{lb}\right)/\left(\varepsilon_{ub}-\varepsilon_{lb}\right)$ is a normalized change in elastin porosity over the experimental range (with lower and upper bounds $\varepsilon_{lb}=0.115$ and $\varepsilon_{ub}=0.699$, respectively), noting that non-dilated aortas in C1041G/+ mice also have nonzero porosity (fig. 2 in [13]). We constrain the maximum reduction of elastic fibre integrity to be $\vartheta_{c^{e}}^{max}$, estimated from the histological data based on the fold change in non-void space (1−ε), that is, $\vartheta_{c^{e}}^{max}=\left(\varepsilon_{ub}-\varepsilon_{lb}\right)/\left(1-\varepsilon_{lb}\right)=0.660$.

Given that a proportion of the elastic fibres in the aorta belong to inter- and intra-lamellar fibril populations, which are thought to play key roles in cell–matrix interactions, we also considered accompanying insults in cellular mechanosensing $\left(\vartheta_{\delta }\right)$, corresponding to a decreased ability of smooth muscle cells to sense intramural stress through integrin binding, as well as mechanoregulation ($\vartheta_{G^{c}}$), consisting of altered pre-stretching of newly deposited collagen via integrins and cytoskeletal activity [16]. We parametrized the evolution of these insults as

(2.6) $$\vartheta_{\delta }=\frac{e^{-g\bar{\varepsilon }}-1}{e^{-g}-1}\vartheta_{\delta }^{max}\;\text{and}\;\vartheta_{G^{c}}=\frac{e^{-g\bar{\varepsilon }}-1}{e^{-g}-1}\vartheta_{G^{c}}^{max},$$

where $\vartheta_{\delta }^{\text{max}}$ and $\vartheta_{G^{c}}^{\text{max}}$ are maximal mechanosensing and mechanoregulation insults, respectively, and g>0 is a constant controlling the insult progression rate relative to that of elastin porosity, approaching a linear relationship with lower values of *g*. Specifically, $\vartheta_{\delta }$ involves increases in the parameter δ from 0 to $\vartheta_{\delta }^{\text{max}}$, and $\vartheta_{G^{c}}$ involves percent reductions in the baseline value of collagen deposition stretch $G^{c}$ bounded by $\vartheta_{G^{c}}^{max}$, as the elastin porosity increases. Equation (2.6) discriminates between independent contributors to aortic aneurysm driven by elastin degradation that can progress along different time scales (insult evolutions are shown in electronic supplementary material, appendix D).

To simulate aneurysmal dilatation, it is useful to retain within the computational domain regions that undergo homeostatic remodelling in contrast to pathological remodelling driven by the prescribed insult. As in previous studies [15,27], we defined spatially heterogeneous profiles for each defect (diminished elastic fibre integrity, dysfunctional mechanosensing and dysfunctional mechanoregulation), varying along the axial direction *z* according to the expression

(2.7) $$\vartheta \left(z_{o}\right)=\vartheta_{\text{apex}}exp\left(-{\left|\frac{z_{o}-l_{o}/2}{z_{od}}\right|}^{v_{z}}\right),$$

where $z_{o}\in \left[0,l_{o}\right]$, with parameters $z_{od}$ and $v_{z}$ governing the axial width and boundary softness of the insult region, respectively, and $\vartheta_{\text{apex}}$ the maximum value of the contributor ($\vartheta_{c^{e}}^{max}$, $\vartheta_{\delta }^{max}$ and / or $\vartheta_{G^{c}}^{max}$) at the insult apex, here centred along the axial length ($z_{o}=l_{o}/2$). We set $z_{od}=3.0\;mm$ and $v_{z}=2$ to achieve an insult region approximately the same size as the experimental ascending aorta dimensions [13] that decayed to baseline values at the model boundaries ($z_{0}=0,l_{o}$), which were fixed in the axial direction. The model length $l_{o}$ was set to 15.0 mm to alleviate any boundary effects. Examples of these insult profiles are shown in the supporting information of [15], Figure G.

### (d) Computational methods

#### (i) Finite-element modelling framework

We have shown that this constrained mixture framework can be simplified via the assumption of mechanobiological equilibrium, in which the heredity integrals can be pre-integrated [27], resulting in a time-independent method for calculating a sequence of G&R responses in a fast and efficient manner using finite elements. We used the open source code FEBio running a custom material plugin to accomplish this computational implementation [15,27]. Briefly, the non-dilated *Fbn1*^*C1041G/*+^ aorta was modelled at the tissue level as a uniform thick-walled cylinder (initial inner radius *a_o_*, thickness *h_o_*, length *l_o_*) to emulate the biaxial experimental set-up. The domain was meshed using 27-node fully integrated quadratic hexahedral elements, with 1 × 20 × 20 elements along the radial, circumferential and axial directions, respectively. Each simulation was initialized with a pre-loading step consisting of axial pre-stretching and luminal pressurization to a systolic value of 120 mmHg to establish an initial *in vivo* state. Next, the evolved, equilibrated G&R state was computed with fixed pressure and a prescribed mechanobiological insult (informed by the elastin porosity values), gradually applied over 10 simulation steps (note that G&R processes tend to occur over weeks). Following the G&R computation, we then fixed the G&R state and adjusted the intraluminal pressure to a diastolic value of 80 mmHg to simulate a cardiac cycle (occurring over the span of milliseconds in mice).

To compute the evolved equilibrated state, it proves convenient to specify the ratio between shear and intramural gain parameters for smooth muscle and collagen ($K_{\tau_{w}}^{m,c}/K_{\sigma }^{m,c}$), as well as the ratio of smooth muscle-to-collagen turnover ($\eta =k_{o}^{m}K_{\sigma ,\tau_{w}}^{m}/k_{o}^{c}K_{\sigma ,\tau_{w}}^{c}$), rather than assigning individual values for each [15,27]. Note that $K_{\tau_{w}}/K_{\sigma }\to 0$ captures a loss of sensitivity in constituent turnover to changes in wall shear stress, and increased η corresponds to increased extracellular collagen production relative to degradation, where the ‘growth-feedback parameter’ for collagen $k^{c}/K_{\sigma ,\tau_{w}}^{c}$ is decreased [15]. It appears that the densities of both smooth muscle cells and endothelial cells decrease in the dilating Marfan aorta [13], noting that endothelial dysfunction can also affect matrix turnover rates, since endothelial derived nitric oxide and endothelin-1 are both potent vasoregulators and influencers of matrix production by smooth muscle cells and fibroblasts. Furthermore, it is well known in Marfan syndrome that both proliferation and apoptosis change dramatically [37,41], hence changing the rates of production and removal. Thus, within the insult region, we reduced the shear-to-intramural gain ratio to $K_{\tau_{w}}/K_{\sigma }=0$ and adjusted the smooth muscle-to-collagen turnover ratio to η=1 in the centre of the vessel according to equation (2.7); values for these parameters at the vessel ends were determined to maintain the properties of the non-insult regions close to the initial state [15]. Additionally, axial-to-circumferential reorientation of the deposition angle for newly deposited collagen was governed by the relation $tan\left(\alpha_{0h}\right)={\left(\lambda_{\theta h}/\lambda_{zh}\right)}^{\gamma }tan\left(\alpha_{00}\right)$, where $\lambda_{\theta h}$ and $\lambda_{zh}$ are stretches in the circumferential and axial directions, respectively; since only modest reorientation was observed in the experimental data [13], we set γ=0.2, allowing small changes in orientation.

**Figure 1.**
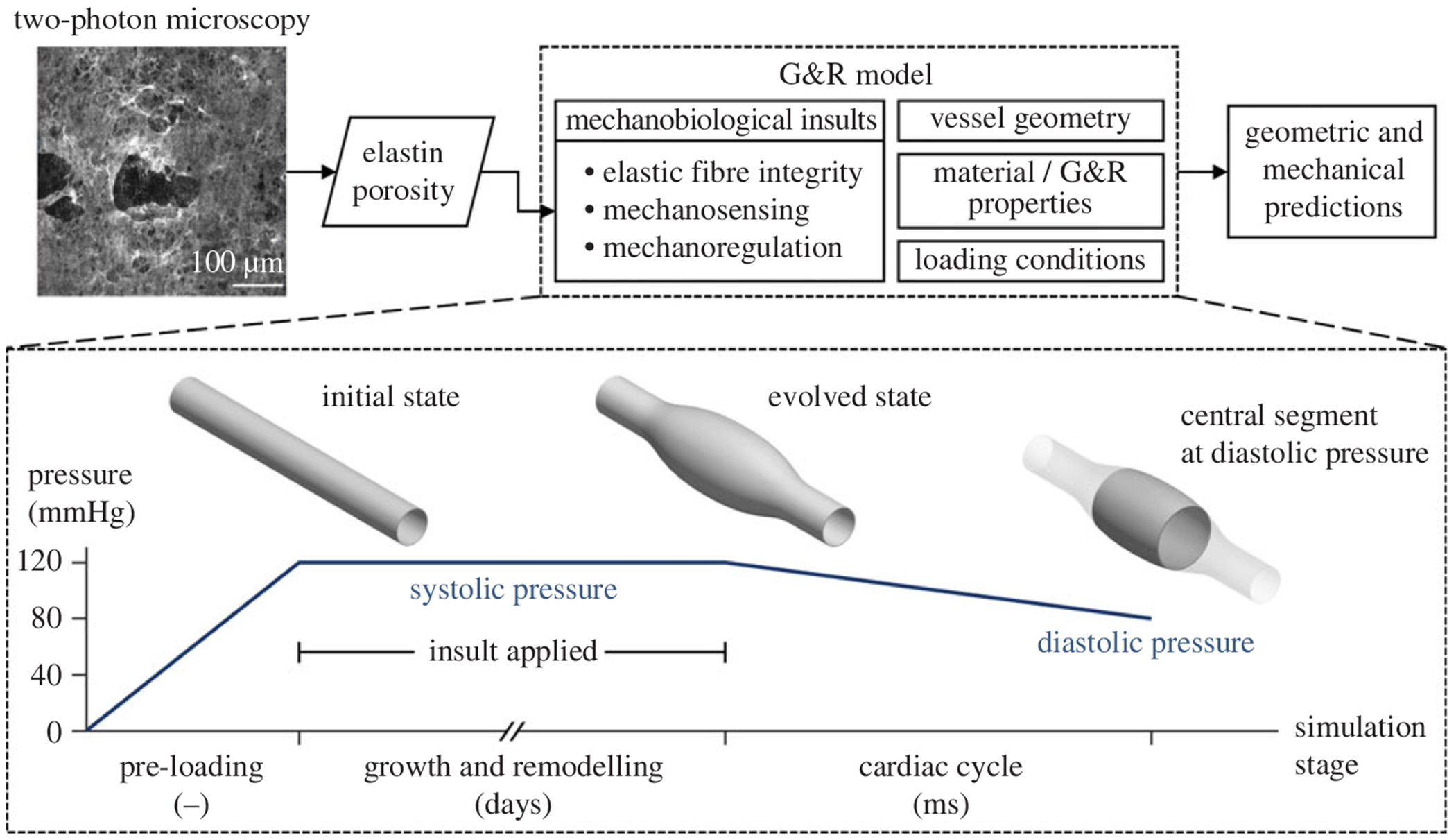
Schema showing the simulation framework. Two-photon microscopy images are post-processed to compute elastin porosity. For each value of porosity, a growth and remodelling (G&R) finite-element simulation is run with an initially uniform cylindrical vessel geometry, fitted material and G&R parameters, *in vivo* loading conditions and corresponding mechanobiological insults affecting elastic fibre integrity, mechanosensing and mechanoregulation. The vessel geometry is first axially pre-stretched and loaded to a systolic value to give the initial state. The insult is then applied incrementally over multiple G&R steps to compute the evolved state. Finally, G&R is arrested, the pressure is reduced to a diastolic value, and geometric and mechanical metrics are evaluated for the central segment of the vessel (at the insult apex).

To facilitate comparison with experimental measurements, geometric and mechanical metrics including luminal diameter, thickness, circumferential and axial stiffness, stored energy and distensibility were evaluated in the central segment of the vessel, corresponding to the experimental domain and the region of maximum luminal diameter. The modelling approach is summarized in figure 1.

#### (ii) Optimization framework

Considering the multiple potential structural and mechanobiological insults related to progressive elastin degradation, we used an optimization approach to determine respective contributions of each factor. Let *Y*(**A**) represent a quantity of interest (e.g. circumferential stiffness) corresponding to insult parameters $A=\left[\vartheta_{\delta }^{max},\vartheta_{G^{c}}^{max},g\right]$, from which we define a normalized mean squared error $J_{Y}$ of the form

(2.8) $$J_{Y}(A)=\frac{1}{n}\sum_{i=1}^{n}{\left(\frac{\left(Y(A)-Y_{h}\right)}{Y_{WT}}\right)}_{i}^{2},$$

where *n* is the number of experimental data points, $Y_{h}$ is the target experimental value, and $Y_{\text{WT}}$ is a normalization term (here, the wild-type group-averaged experimental measurements). Note that the parameter $\vartheta_{c^{e}}^{max}$ was excluded from optimization because the insult in elastic fibre integrity was defined based on experimental measurements of elastin porosity. Values for *Y* were calculated for all elastin porosities over the experimental range, with the different insults defined by equations (2.5) and (2.6).

**Figure 2.**
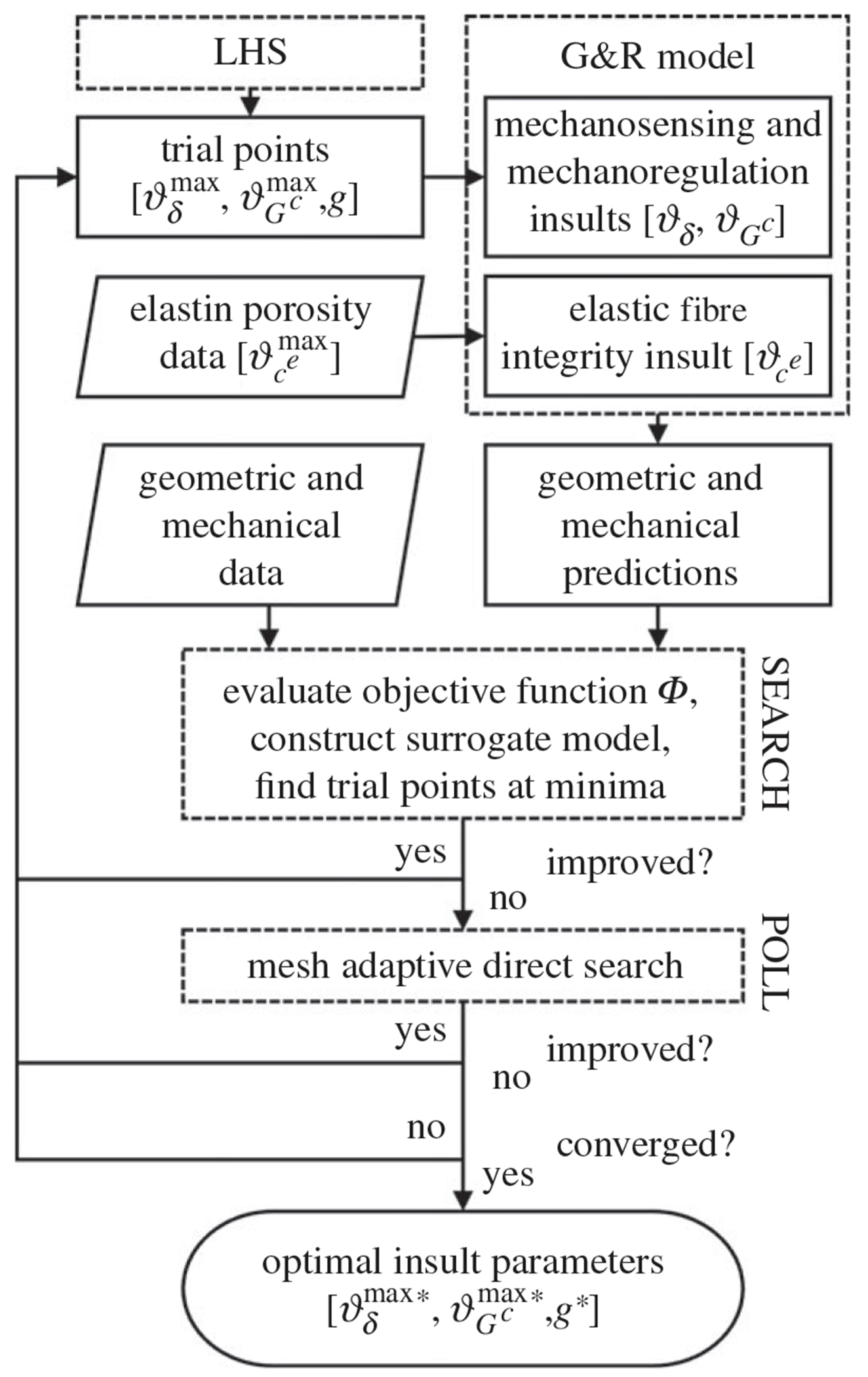
Schema illustrating the optimization algorithm using a surrogate management framework. Latin Hypercube Sampling (LHS) is used to generate an initial parameter set $\left[\vartheta_{\delta }^{max},\vartheta_{G^{c}}^{max},g\right]$ for mechanosensing and mechanoregulation insults $\left[\vartheta_{\delta },\vartheta_{G^{c}}\right]$, whereas experimental elastin porosity measurements are used to define the range ($\vartheta_{c^{e}}^{max}$) of elastic fibre integrity insult ($\vartheta_{c^{e}}$). Geometric and mechanical metrics from experimental data (parallelograms) and G&R model predictions are compared to evaluate the objective function Φ. In the SEARCH step, a surrogate model is constructed from objective function evaluations using kriging, and trial points at local minima of the surrogate are evaluated. In the subsequent POLL step, a mesh adaptive direct search is used to find an improved point. The optimal insult parameters $\left[\vartheta_{\delta }^{max*},\vartheta_{G^{c}}^{max*},g^{*}\right]$ are identified once the minimum is found and convergence criteria are fulfilled.

Experimental quantities of interest included both geometric (inner diameter and wall thickness) and mechanical (stored energy, circumferential and axial stiffness, distensibility) metrics. Given previous work identifying circumferential stiffness $c_{\theta \theta \theta \theta }$ and stored energy *W* as key indicators of aortic function [42], we used these as the basis for the objective function *Φ* used to minimize differences between experimentally computed and model-predicted measures:

(2.9) $$\Phi =\sqrt{\frac{\left(J_{c_{\theta \theta \theta \theta }}(A)+J_{W}(A)\right)}{2}},$$

and optimal insult parameters $A^{*}$ were obtained via minimization of Φ, accomplished using a derivative-free surrogate management framework algorithm [43]. Briefly, Latin Hypercube Sampling was used to obtain an initial set of trial points for evaluations of the objective function. Kriging was then used to construct an interpolated surface in the parameter space whose minima are identified during a SEARCH step. Once a minimum has been identified, candidate points around the minimum are then evaluated in a POLL step with a mesh adaptive algorithm to find an improved value. SEARCH steps are repeated whenever an improved point is found, and the search terminates once SEARCH and POLL steps fail to return improved minima and the mesh refinement threshold is reached. Figure 2 represents the optimization framework schematically, and table 2 shows the bounds on the insult parameters explored herein. For each insult contributor $\vartheta_{\delta ,G^{c}}^{max}$, a lower bound of 0 corresponds to absence of the effect; the upper bound was chosen within the range of previous *in silico* investigations [15] to ensure model stability.

## 3. Results

### (a) Performance of individual insult contributors

**Figure 3.**
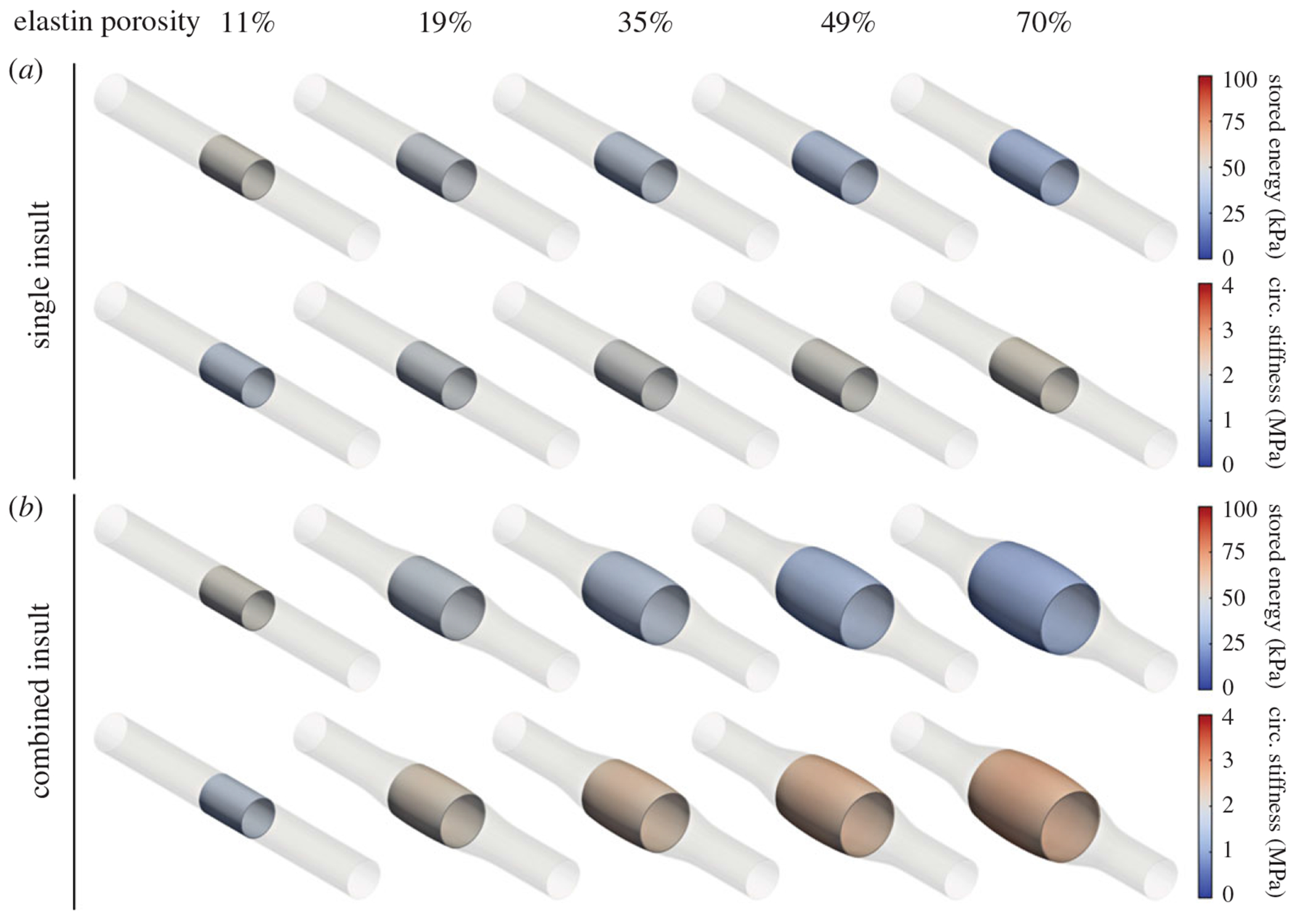
Evolution of model-predicted vessel geometry and mechanical properties (stored energy and circumferential stiffness) as a function of increasing elastin porosity (11–70%) left-to-right for (*a*) insult defined by a single contributor, diminished elastic fibre integrity (0–66%) and (*b*) combined insult consisting of diminished elastic fibre integrity (0–66%) plus dysfunctional mechanosensing (0–6.7%) and mechanoregulation (0–0.72%). The vessel has baseline properties *W_0_* = 51.9 kPa and *c*_*θθθθo*_ = 1.52 MPa. The central segment near the insult apex, representing the ascending aorta, is highlighted for clarity.

**Figure 4.**
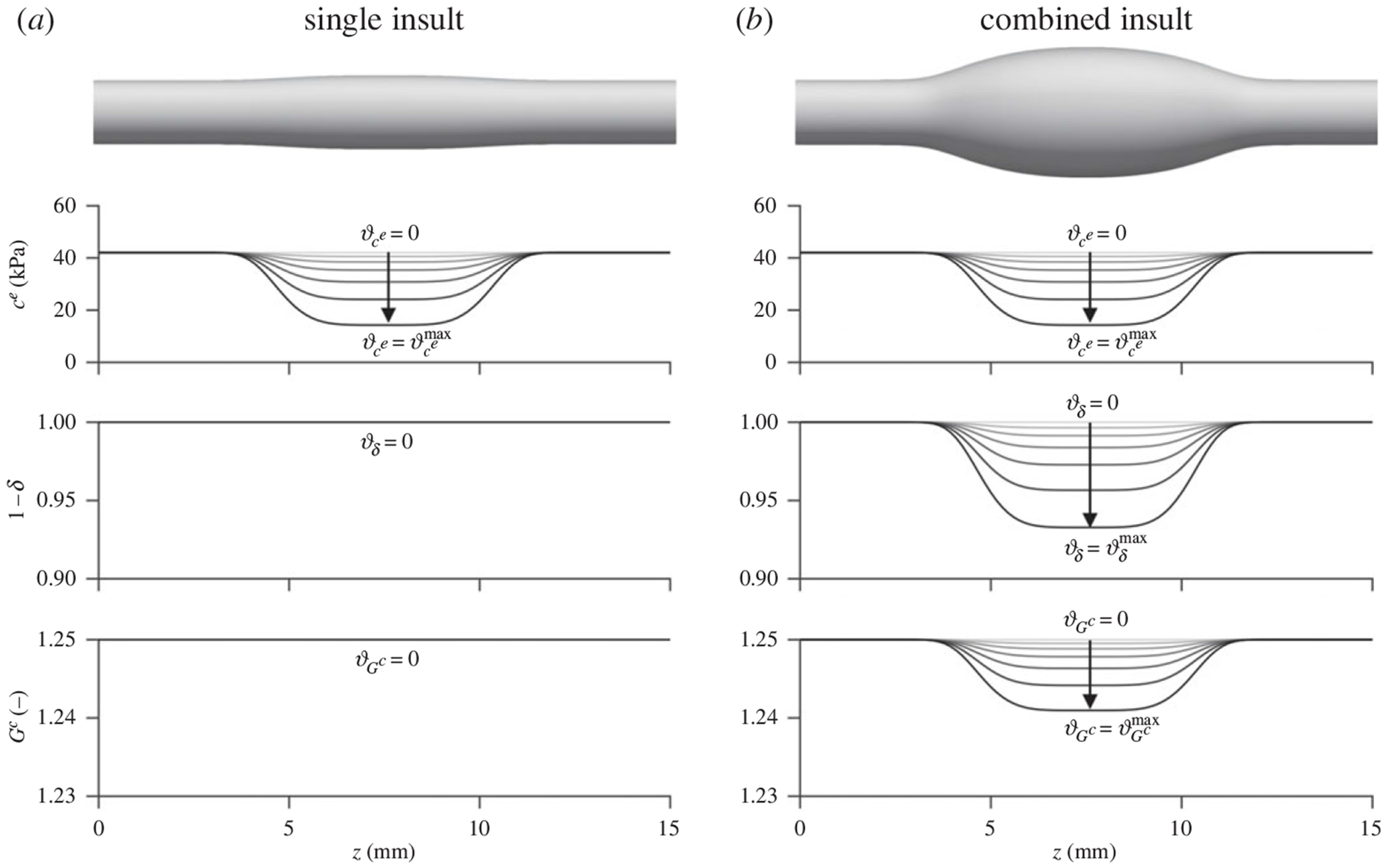
Axial variation of mechanobiological insults (according to equation (2.7)) for (*a*) single and (*b*) combined insult models. Top-to-bottom: elastin modulus *c*^*e*^, reduction driven by $c^{e}=0.660$, dysfunctional mechanosensing (increased *δ*), driven by $\vartheta_{c^{e}}=0.067$, and dysfunctional mechanoregulation (decreased collagen deposition stretch $G^{c}$), driven by $\vartheta_{G^{c}}=0.0072$. For all plots, insult progression from lightest to darkest lines is analogous to the simulation results shown in figure 3 from left to right. For all simulations, baseline values at *z* = 0, 15 mm are listed in table 1, and smooth muscle-to-collagen turnover ratio *η* and shear-to-intramural gain ratio $K_{\tau_{w}}/K_{\sigma }$ vary with similar axial profiles.

Given microstructural data that show an increased porosity of elastic fibres in Marfan syndrome, we initially focused on characterizing the effect of structural insults to elastic fibre integrity alone. Simulations were run for porosity values ranging from approximately 11 to 70%, corresponding to diminished elastic fibre integrity ($\vartheta_{c^{e}}$) from 0–66% (figure 3a), in which the luminal diameter, stored energy and circumferential stiffness were evaluated in the central segment of the model (insult profile shown in figure 4a) at diastolic pressure. Recall that simulations with compromised elastic fibre integrity alone were not based on fits to the experimental data since this insult was estimated from elastin porosity measurements; rather, these simulations were intended to bracket the range of geometric and mechanical responses arising from elastin damage and degradation. Isolated insults to elastic fibre integrity accurately captured the decrease in stored energy (52 kPa decreasing to 27 kPa) relative to that of the *Fbn1*^*C1041G/*+^ and *Fbn1^mgR/mgR^* groups observed experimentally (48 kPa decreasing to 29 kPa). However, this insult model did not reproduce the dramatic increases in circumferential stiffness (2.2 MPa predicted versus 3.2 MPa observed experimentally, cf. fig. 6 in [13]).

Next, we performed similar simulations for dysfunctional mechanosensing alone (with parameter $\vartheta_{\delta }$ from 0 to 0.150) and dysfunctional mechanoregulation alone (with parameter $\vartheta_{G^{c}}$ from 0 to 0.012), shown in electronic supplementary material, appendix E. These models produced much milder changes in stored energy than that observed with insults to elastic fibre integrity; indeed, insults in mechanosensing alone resulted in modestly increased stored energy, while insults in mechanoregulation had little effect. Additionally, neither insult achieved sufficient circumferential stiffening, particularly for losses in mechanoregulation.

By contrast, insults combining diminished elastic fibre integrity with concomitant dysfunctional mechanosensing and mechanoregulation (figure 4b) maintained a reduced stored energy comparable to that of the elastic fibre insult alone while predicting significantly more circumferential stiffening (2.8 MPa) than individual insults (figure 3b). Based on these findings, it appeared that a combined insult consisting of diminished elastic fibre integrity and dysfunctional mechanosensing and mechanoregulation of matrix was required to recapitulate the trends in mechanical properties observed in the experimental data.

### (b) Combined insult model captures geometric and mechanical properties of aortic aneurysm

**Figure 5.**
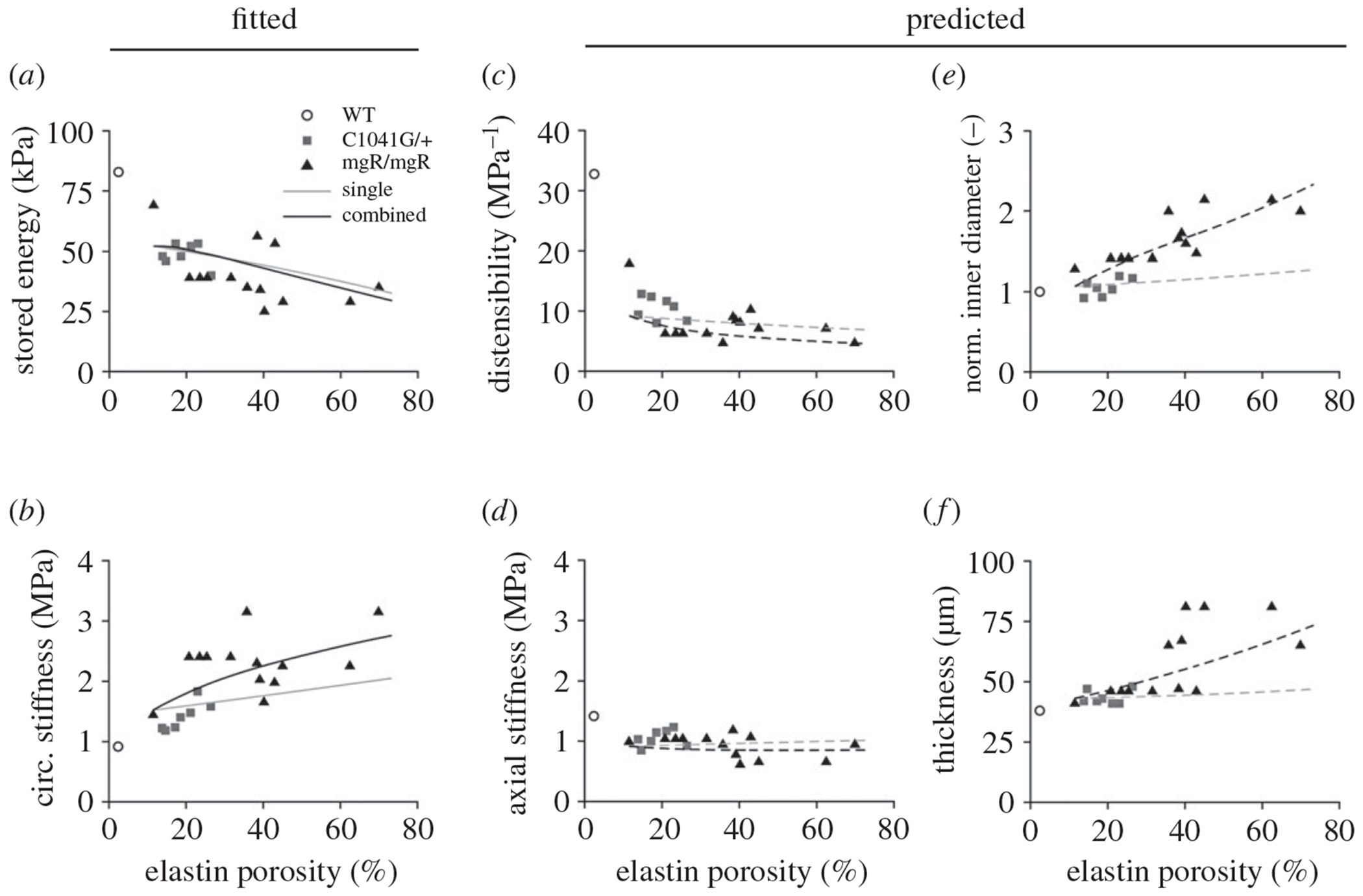
Model predictions for vessel geometry and mechanical properties at diastolic pressure (80 mmHg) plotted against elastin porosity. Experimental data (symbols) include measurements of (*a*) stored energy, (*b*) circumferential stiffness, (*c*) distensibility, (*d*) axial stiffness, (*e*) normalized inner diameter, and (*f*) thickness from *Fbn1*^*C1041G/*+^ (grey squares) and *Fbn1*^*mgR/mgR*^ (black triangles) groups; adapted from [13]. Model predictions (lines) for metrics in the central segment (figure 3) are compared for simulations involving a single insult contributor (elastic fibre integrity only, grey lines) and combined insults with compromised elastic fibre integrity and dysfunctional mechanosensing and mechanoregulation (black lines). Solid lines indicate model fits to the data (*a*,*b*), and dashed lines indicate fitted model predictions (*c*–*f*). Note that the wild-type data (WT, open circles) were not used in the optimizations or simulations; they are shown merely as reference values, emphasizing that a non-dilated baseline Marfan aorta is yet different from normal.

Recall that our optimization of best-fit model parameters sought to minimize differences between experimentally measured and computationally simulated stored energy and circumferential stiffness at diastolic pressure, two metrics that have been reported to be effective predictors of aortic function in murine aneurysm models [42]. The combined insult model fit the experimental data well, with diminished elastic fibre integrity driving the reduced stored energy and the addition of dysfunctional mechanosensing and mechanoregulation driving the increase in circumferential stiffness at higher values of elastin porosity (figure 5a,b).

Additional metrics, including distensibility, axial stiffness, normalized inner diameter (ratio relative to wild-type) and wall thickness, were then predicted using the fitted model. The efficacy of considering combined mechanical and mechanobiological effects was further highlighted in the enhanced predictive capability whereby we observed improved predictions in decreasing distensibility and axial stiffness along with increasing luminal diameter and thickness (figure 5c–f). Simulations with elastic fibre integrity insults alone appeared to provide reasonable predictions of distensibility and axial stiffness; however, they underestimated the experimentally measured increases in inner diameter and wall thickness as elastin porosity increased.

### (c) Link between elastin porosity and mechanobiological factors

Optimization was also performed over multiple independent trials to assess the stability of the best-fit cell-mediated insult parameters [$\vartheta_{\delta }^{max*}$, $\vartheta_{G^{c}}^{max*}$, $g^{*}$] (table 2). While the value for $g^{*}$ was relatively consistent over multiple optimization trials, the mechanosensing and mechanoregulation contributions ($\vartheta_{\delta }^{max*}$ and $\vartheta_{G^{c}}^{max*}$, respectively) varied considerably, despite achieving good fits in all cases. Placed in the context of previous *in silico* studies of insults to mechanosensing and mechanoregulation in a wild-type aorta, the average value for mechanosensing resulting from optimization tended toward the ‘mild’ end of the explored range, whereas that for mechanoregulation tended toward the ‘severe’ end (cf. figs. 1 and 2 in [15]). The high average value of the progression rate also suggested that the optimal combined insult involved a dysfunctional mechanosensing and mechanoregulation that progressed more quickly relative to the diminishing elastic fibre integrity at mild elastin porosities, decaying to a more gradual loss at severe porosities (that is, g* <5 would suggest a more linear relationship between elastin porosity and mechanosensing/regulation insults, see electronic supplementary material, appendix F).

### (d) Correlations between cellular mechanosensing and mechanoregulation

Statistically significant correlations between the maximum insult values and the progression rate were also observed, with mechanosensing inversely related to both mechanoregulation and progression rate (plots of optimal parameters with respect to one another are shown in electronic supplementary material, appendix G). Recall that in the constrained mixture framework, cellular mechanosensing and mechanoregulation of matrix enter the formulation as distinct mechanisms, with mechanosensing affecting the detected deviation in intramural stress Δσ that contributes to mass density production and removal (equation (2.3)) and mechanoregulation affecting the stored energy term for newly deposited collagen. Despite these distinctions, it appeared that mechanosensing and mechanoregulation had similar tissue-level consequences.

## 4. Discussion

The primary function of the aorta is to serve as an efficient elastic conduit for transporting blood from the left ventricle to medium-sized arteries that distribute the blood to tissues throughout the body. This function is achieved, in part, by storing elastic energy during systolic distension and using this energy to work on the blood to augment flow during diastolic recoil. Competent elastic fibres endow the normal aorta with its ability to distend (compliance) and recoil (resilience) while fibrillar collagens endow the wall with stiffness and strength. It has long been known that progressive aneurysmal dilatation associates with both diminished elastic fibre integrity and remodelling of collagen fibres [44–46], recently examined further in a mouse model that combines elastase exposure to compromise elastic fibres with *β*-aminopropionitrile to block the deposition of competent collagen fibres during remodelling [36]. Whereas a progressive increase of elastin porosity is commonly observed histologically [12], progressive remodelling of fibrillar collagen is more difficult to quantify [13], particularly given its rapid turnover. Computational models offer the distinct advantage of assessment of diverse cell-mediated contributors to aneurysmal dilatation, including loss of elastic fibre integrity [36,46], collagen remodelling [44,45,47,48], and cellular mechanosensing and mechanoregulation [15].

There have been few detailed computational studies of G&R in TAAs, most based on limited human data [49,50], due in part to a lack of consistent histological and biomechanical data. In this paper, we used a computationally efficient implementation of a constrained mixture model of G&R [27] to test hypotheses regarding roles of isolated and combined insults known to drive TAAs. Toward this end, we used recent data collected in our laboratory that compared biaxial aortic properties in wild-type and the two most common models of Marfan mice [13], and employed a formal method of optimization [43] to capture trends in the Marfan data specifically. Parametrizing for the mechanical properties of the *Fbn1*^*C1041G/*+^ aorta as a baseline, we observed different behaviours compared with those of healthy wild-type aortas [15] in response to localized defects that can give rise to progressive aortic dilatation; in fact, the model could only describe the actual data when multiple disease-contributing factors coexisted. Moreover, determination of an optimal combined insult required an initial rapid progression of contributing mechanobiological factors that reduced in rate as elastin porosity increased, consistent with an ‘accelerated vascular ageing’ description [51]. That effects of a mutation to a single gene (*Fbn1* in mice) are manifold is to be expected, given that fibrillin-1 microfibrils not only contribute to elastic fibre homeostasis, they also promote smooth muscle cell mechanosensing of matrix and affect the bioavailability of different biomolecules, including transforming growth factor *β*, which can affect mechanoregulation of matrix via changes in both the production of extracellular matrix and elaboration of contractile proteins. Our computational findings are thus consistent with myriad effects of compromised fibrillin-1 in the Marfan aorta [52].

**Figure 6.**
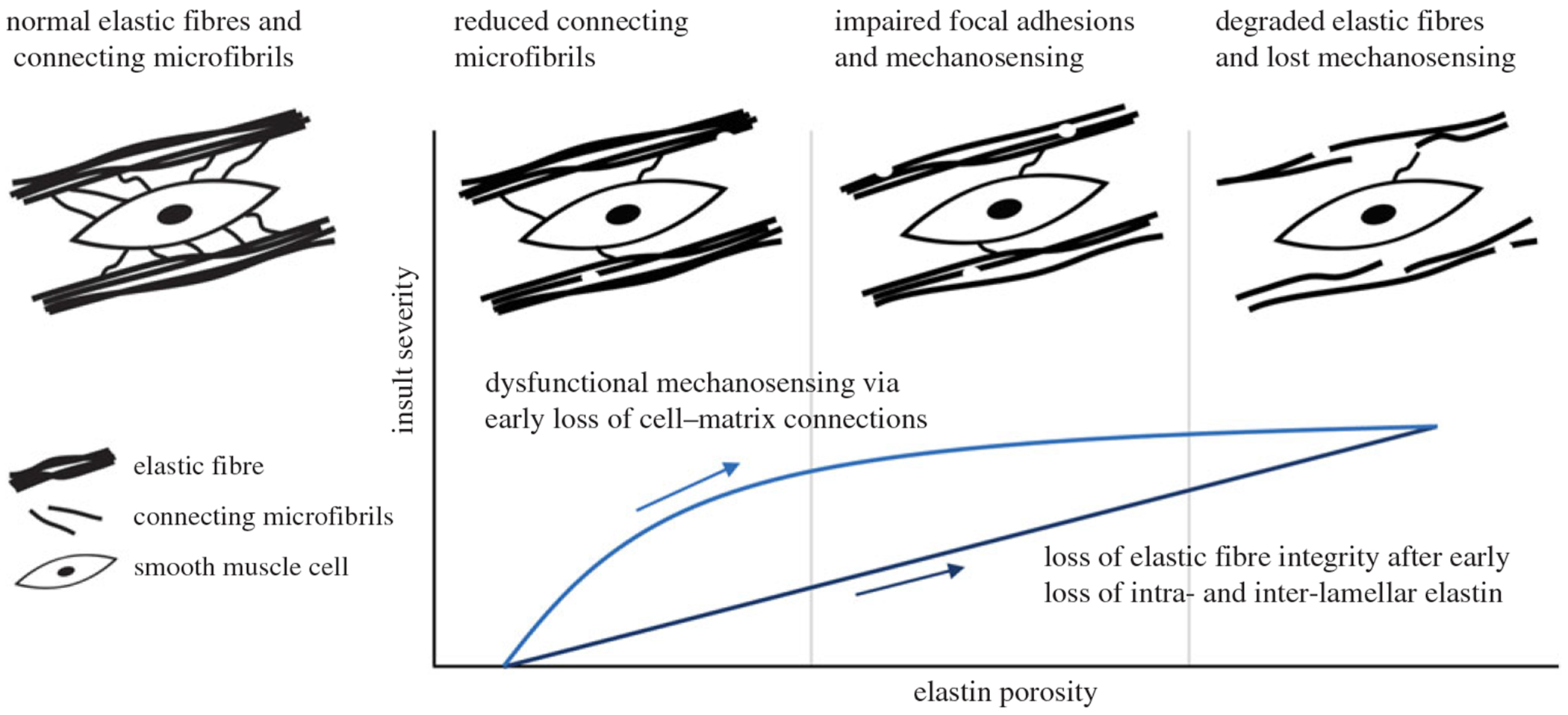
Conceptual model for dysfunctional mechanosensing associated with increasing elastin porosity in Marfan syndrome. Smooth muscle cells in the normal media are anchored in maturity to elastic lamellar structures via inter- and intra-lamellar connecting microfibrils through which they mechanosense the surrounding matrix. As elastin porosity increases with progressive deterioration of the Marfan aorta, cell focal adhesions are impaired as connecting microfibrils are lost, thus compromising cellular mechanosensing (and likely mechanoregulation). Loss of elastic fibre integrity (i.e. damage to and degradation of elastic lamellae) may progress at a comparably reduced rate.

There is, however, a need for continued investigation. At the tissue scale, it remains difficult to delineate the interplay between cellular mechanosensing and mechanoregulation, as seen in the correlations between the optimal insult parameters over multiple trials. Compromised mechanosensing has been conjectured based on the diverse mutations that predispose to TAAs [16] and is supported by the observed loss of intra- and inter-lamellar elastin [53] and recent studies showing altered integrin signalling in the Marfan aorta [37–39]. These diverse observational and experimental results are now supported by our computational results herein. Indeed, the nonlinearity of insult progression emerging from the present optimization offers a quantitative description of reduced cellular mechanosensing in which connecting elastic filaments may degrade before the highly cross-linked elastic fibres that constitute the lamellar structures (figure 6). Experimental data supporting the possibility of compromised mechanoregulation of extracellular matrix will be challenging to identify—it was even suggested in the original proposal of the constrained mixture model [23] that it would not be possible to infer deposition stretches directly from data—rather, their roles would best be evaluated on the basis of associated consequences. The present work supports this suggestion, showing that dysfunctional mechanoregulation of matrix can play a strong contributing role in aneurysmal dilatation. Recent studies including RNA sequencing have yet revealed that hundreds of genes are differentially expressed in Marfan mice [54–56], some of which are easily imagined to reflect dysfunctional mechanosensing and mechanoregulation of matrix (particularly via reductions in smooth muscle cell contractile genes). Toward this end, models coupling tissue-level and microstructural biomechanics with cell signalling (e.g. [57]) should be pursued to better understand relationships between transcript and tissue. There is also a need to examine theoretically in more detail the roles of individual contributors to aortic integrity that promote its mechanobiological stability [26] or its apparent loss when aneurysmal [24].

We have also observed through analysis of standard histological sections stained with Movat’s pentachrome that a substantial amount of glycosaminoglycans (GAGs) accumulates within the media as elastin degrades (see electronic supplementary material, appendix A). While we have modelled this accumulation previously [36], potential associated effects were not included in this three-dimensional model. Despite this, GAGs are not expected to influence the tensile properties (that is, material stiffnesses) of the Marfan aorta; thus, it appears that mechanosensing and mechanoregulation will remain as central candidates in our search to understand mechanisms by which aneurysmal dilatation and associated circumferential stiffening occur. Further aspects including separate modelling of medial and adventitial layers in the aortic wall, as well as spatially varying vessel material properties between the inner and outer curvature of the ascending aorta, will also need to be included. With novel microstructural data collected to this end [13], as well as advances in quantifying regional variations in mechanical properties [36], it will become increasingly important to capture these effects in future G&R studies.

## 5. Conclusion

In closing, aneurysms of the thoracic aorta that progress to dissection and rupture are increasingly responsible for significant morbidity and mortality. Even in cases of monogenic mutations predisposing to these lesions (e.g. in Marfan syndrome), there is a need to understand better the broad effects of differential gene expression, including delineation of pathological consequences and protective compensations. The present computational results show quantitatively for the first time the importance of multiple simultaneous contributors to TAA progression, yet continued studies using multiscale computational models promise to contribute further to this understanding and must continue to be pursued.

## Supplementary Material

Electronic supplementary material

Electronic supplementary material is available online at https://doi.org/10.6084/m9.figshare.c.6760123.

## Figures and Tables

**Table 1.** Baseline material and G&R parameters for Marfan mouse ascending aortas prior to aneurysmal development. Superscripts *e*, *m*, *c* denote elastin-dominated matrix, smooth muscle and collagen-dominated matrix, respectively; super/subscripts *r*, *θ*, *z*, *d* denote radial, circumferential, axial and symmetric diagonal directions, respectively. Different material behaviours result from different values of the collagen material parameters in equation (2.4), illustrated in electronic supplementary material, appendix C. In this study, elastin, collagen and mechanosensing parameters were evolved with prescribed insults based on elastin porosity.

fixed	parameter	value
inner radius, thickness	$a_{0},h_{0}$	0.809 mm, 0.041 mm
elastin, smooth muscle, collagen mass fractions	$\phi_{0}^{e},\phi_{0}^{m},\phi_{0}^{c}$	0.354, 0.345, 0.311
collagen orientation fractions	$\beta^{\theta },\beta^{z},\beta^{d}$	0.012, 0.012, 0.976
smooth muscle material parameters	$c_{1}^{m},c_{2}^{m}$	1.26 kPa, 30.0
collagen material parameters	$c_{1}^{c},c_{2}^{c}$	665.6 kPa, 2.14
elastin deposition stretches	$G_{r}^{e},G_{\theta }^{e},G_{z}^{e}$	$1/\left(G_{\theta }^{e}G_{z}^{e}\right)$, 1.74, 2.24
smooth muscle and collagen deposition stretches	$G^{m}$	1.19
evolving	parameter	value
elastin material parameter	$c^{e}$	42.03 kPa
collagen deposition stretch	$G^{c}$	1.25
diagonal collagen orientation	$\alpha_{00}$	51.1°
mechanosensing	δ,ξ	0, 0
growth and remodelling	parameter	value
smooth muscle-to-collagen turnover ratio	η	0.9
shear-to-intramural gain ratio	$K_{\tau_{w}}/K_{\sigma }$	0.9

**Table 2.** Optimization bounds for combined insult parameters and results for eight independent runs. $\vartheta_{\delta ,G^{c}}^{max}$ determine the maximal insults in mechanosensing (increased δ ) and mechanoregulation (decreased $G^{c}$), and *g* describes the progression in insult severity $\vartheta_{\delta ,Gc}$ according to equation (2.6) as elastin porosity increases. Note that the maximal insult to elastic fibre integrity $\vartheta_{c^{e}}^{\text{max}}$ (reduced $c^{e}$) was fixed at 0.660 based on direct histological findings.

	mechanosensing $\vartheta_{\delta }^{max*}$	mechanoregulation $\vartheta_{G^{c}}^{max*}$	progression rate g*
bounds	[0, 0.1000]	[0, 0.0150]	[0.01, 10.00]
Trial 1	0.0512	0.0089	6.53
Trial 2	0.0629	0.0077	5.75
Trial 3	0.0950	0.0043	6.00
Trial 4	0.0153	0.0120	7.05
Trial 5	0.0445	0.0094	6.66
Trial 6	0.0672	0.0072	5.88
Trial 7	0.0725	0.0068	6.00
Trial 8	0.0669	0.0074	5.76
mean±s.e.m.	0.0594±0.0092	0.0080±0.0008	6.21±0.170

## Data Availability

The data used in this work have been reported in https://www.frontiersin.org/articles/10.3389/fcvm.2021.800730/full [14]. Source code for the custom FEBio material plugin used for simulating equilibrated growth & remodelling evolutions is openly accessible from the GitHub repository: https://github.com/yale-cbl/mbecmm-febio. The data are provided in electronic supplementary material [58].
