# Supplementary material for: Computational modelling distinguishes diverse contributors to aneurysmal progression in the Marfan aorta
Source: Proc Math Phys Eng Sci. Author manuscript; Available in PMC 2025 Oct 22. (PMC12539648; doi:10.1098/rspa.2023.0116)
Supplement: Electronic supplementary material [file NIHMS2117055-supplement-Electronic_supplementary_material.pdf]

Computational modeling distinguishes diverse contributors to aneurysmal progression in the Marfan aorta

Electronic Supplementary Material

David S. Li, Cristina Cavinato,
Marcos Latorre, Jay D. Humphrey

Appendix A Experimental methods

A.1 Biaxial mechanical testing

Most of the experimental data used herein were reported previously [1], though with some subsequent data collection and analysis to extend the groups. For completeness, note these studies included two groups of 9-week-old male mice: $Fbn1^{C1041G/+}$ (from Jax Mice) and $Fbn1^{mgR/mgR}$ (derived from breeding pairs from Mt. Sinai Ichan School of Medicine), both maintained on a C57BL/6J background. All live animal studies were approved by the Institutional Animal Care and Use Committee of Yale University. Following euthanasia, the ascending thoracic aorta (ATA) was excised from the aortic valve past the brachiocephalic artery and cleaned of perivascular tissue. It was then cannulated with custom-drawn glass micro-pipets through the aortic root and the brachiocephalic artery, with all branches ligated with sutures.

Mechanical testing was carried out within hours of euthanasia using a computer-controlled biaxial device while the specimen remained immersed in a Hanks buffered saline solution at room temperature to ensure passive behaviors. Following equilibration at a distending pressure of 80 mmHg and *in vivo* value of axial extension and preconditioning via four cycles of pressurization from 80 to 140 mmHg, the computer performed seven cyclic loading protocols: three pressure-diameter tests at three different fixed axial stretches (0.95, 1.00, and 1.05 times the subject-specific *in vivo* axial stretch) and four force-length tests at fixed luminal pressures (10, 60, 100, and 140 mmHg). Distending pressure, applied axial force, outer diameter, and axial length were recorded for all cyclic protocols and used to inform a pseudoelastic constitutive model of the passive behavior, consisting of a neoHookean term and a four-fiber family exponential model. Wall thickness was calculated at each pressure-force state under the assumption of incompressibility using measurements in the unloaded configuration taken using a dissecting microscope at the completion of the experimental protocol. A detailed description of the experiments and model calculations can be found elsewhere [2, 3].

A.2 Microstructural analysis

Following biaxial mechanical testing, samples were incubated overnight in a 2-µM fluorescent red nucleic acid stain (SYTO™ 61) solution. The three-dimensional (3D) structure of the principal components of the tissue — collagen, elastin, and all cell nuclei — was characterized using two-photon microscopy (LaVision Biotec TriMScope), operated

with a Ti-Sa Laser (Chameleon Vision II, Coherent) and imaged with an Olympus 20x water immersion objective lens (NA 0.95). The excitation wavelength was tuned to 840 nm, and signal detection was operated simultaneously at three different wavelength ranges: 390–425 nm for second harmonic generation signal from collagen fibers, 500–550 nm for autofluorescence of elastic fibers, and above 550 nm for fluorescence of stained cell nuclei. Image acquisition was in the axial-circumferential plane of the vessels with a field of view of 500 μm $\times$ 500 μm and a 2-μm step in the radial direction, consistently in the same anatomical position and orientation for all ATA samples. Samples were imaged at distending pressures of 80 mmHg and sample-specific *in vivo* axial stretch, after preconditioning with four pressurization cycles between 0 and 140 mmHg. Further information on the imaging protocol and analysis have been previously detailed in [1].

3D images were transformed to cylindrical coordinates and post-processed using ImageJ v1.53 and MATLAB. Intensity profiles along the radial axis were acquired and used to define wall layer-specific positions by automatic identification of internal and external surfaces and the interface between medial and adventitial layers. Elastin porosity was quantified within the media layers as the ratio between the volume identified as voids and the volume occupied by elastic fibers. For this purpose, the mean intensity profile of the elastin signal was used to radially exclude the interlamellar spaces by removing from the quantification the radial planes identified as local minima and their $\pm$1-μm neighbor radial planes. An adaptive three-dimensional filter was applied to the remaining volume to reduce possible inhomogeneities due to local waviness of the elastic lamellae. The filtered image was binarized using the automatic local Phansalkar thresholding method, and the volume of the voids was quantified as a black-to-white ratio.

A.3 Histology

For standard histological examination, fixed specimens were embedded in paraffin and sectioned serially in the radial-circumferential plane with a section thickness of 5 μm. Mounted slides were stained with Movat's pentachrome to detect elastin in black, collagen in yellow/brown, smooth muscle cell and fibroblast cytoplasm in purple, aggregating glycosaminoglycans/proteoglycans (GAGs/PGs) in blue/aqua, and fibrin in red. Slides were imaged at 20x magnification using an Olympus BX/51 microscope under standard bright-field conditions with a $\sim$180-μs exposure time. Standard histology revealed the structure of the elastic lamellae in the media and their extensive fragmentation in severe disease (though not the 3D structure or inter- and intra-lamellar elastin) as well as the loss of cytoplasm and accumulation of GAGs in the media.

We employed a custom color-based analysis script to categorize each pixel (Figure A.1) and digitally extract the medial and adventitial layers of each section for estimation of layer areas. Constituent area fractions were then computed on a layer-specific basis for each group (Table A.1). The evolution of elastin, smooth muscle cell, and collagen constituents from mild to severe elastin degradation was then captured with a model for growth and remodeling of a bilayered artery (Appendix B).

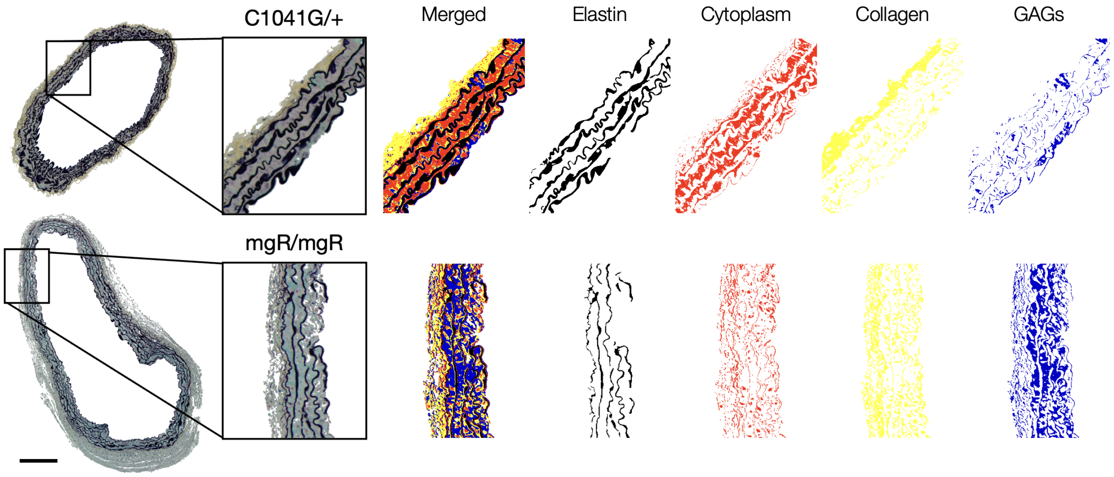

Figure A.1: Histological analysis of representative (top) $Fbn1^{C1041G/+}$ and (bottom) $Fbn1^{mgR/mgR}$ cross-sections stained with Movat's pentachrome. Color-based analysis routine used to categorize image pixels as elastin (black), cytoplasm (red), fibrillar collagen (yellow), glycosaminoglycans (GAGs, blue), and fibrin (absent, thus not shown), digitally separated for clarity. In particular, for these representative samples, note percent changes from the less severe missense to the more severe hypomorphic models: elastin decreased from 34 to 14%, cytoplasm decreased from 26 to 11%, collagen decreased from 29 to 28%, and GAGs increased from 11 to 49%. Scale bar = 200 µm.

Table A.1: Histological quantification for Movat's pentachrome-stained sections from $Fbn1^{C1041G/+}$ (N = 20) and $Fbn1^{mgR/mgR}$ (N = 12) ascending aortas: wall percentages of media and adventitial layers and constituent area fractions for total wall and on a layer-specific basis.

		Wall	Elastin	Cytoplasm	Collagen	GAGs	Fibrin
Total wall	C1041G/+	—	0.354 ± 0.01	0.170 ± 0.01	0.311 ± 0.01	0.165 ± 0.01	0.0 ± 0.0
	mgR/mgR	—	0.233 ± 0.03	0.143 ± 0.01	0.318 ± 0.01	0.304 ± 0.03	0.0 ± 0.0
Media	C1041G/+	0.727 ± 0.01	0.486 ± 0.02	0.197 ± 0.01	0.105 ± 0.01	0.213 ± 0.01	0.0 ± 0.0
	mgR/mgR	0.692 ± 0.02	0.320 ± 0.04	0.172 ± 0.01	0.178 ± 0.01	0.329 ± 0.04	0.002 ± 0.001
Adventitia	C1041G/+	0.273 ± 0.01	0.004 ± 0.0	0.093 ± 0.003	0.861 ± 0.004	0.042 ± 0.00	0.0 ± 0.0
	mgR/mgR	0.308 ± 0.02	0.004 ± 0.001	0.087 ± 0.01	0.652 ± 0.04	0.257 ± 0.04	0.0 ± 0.0

Appendix B Model parameterization

We previously estimated the parameters of a four-fiber family constitutive model fitted to the group-averaged biaxial data of the $Fbn1^{C1041G/+}$ and $Fbn1^{mgR/mgR}$ groups [1]. Synthetic data were then generated for six protocols (three pressure-diameter tests at 0.95, 1, and 1.05 times the *in vivo* axial stretch and three force-length tests at fixed luminal pressures of 60, 100, and 140 mmHg) using an average unloaded geometry for each group.

Next, a bilayered rule-of-mixtures material model with growth and remodeling (G&R) [4] was fit to the synthetic data from both groups to capture changes from less severe ($Fbn1^{C1041G/+}$) to more severe ($Fbn1^{mgR/mgR}$) disease states. Estimated constituent fractions in the media and adventitia computed from histology were incorporated to compute

constituent-specific stored energy parameters as well as deposition stretches for elastin, smooth muscle, and collagen. In particular, parameters for elastin mass and deposition stretch and collagen fiber family fractions and orientations were fixed, whereas elastin, smooth muscle, and collagen stored energy parameters and smooth muscle and collagen deposition stretches were allowed to evolve. We used a nonlinear least squares regression algorithm in MATLAB to estimate best-fit values, and the parameters capturing the $Fbn1^{C1041G/+}$ data are reported in Table 1. The fitted model results are shown in Figure B.1, and further details are described in the Appendix of [4].

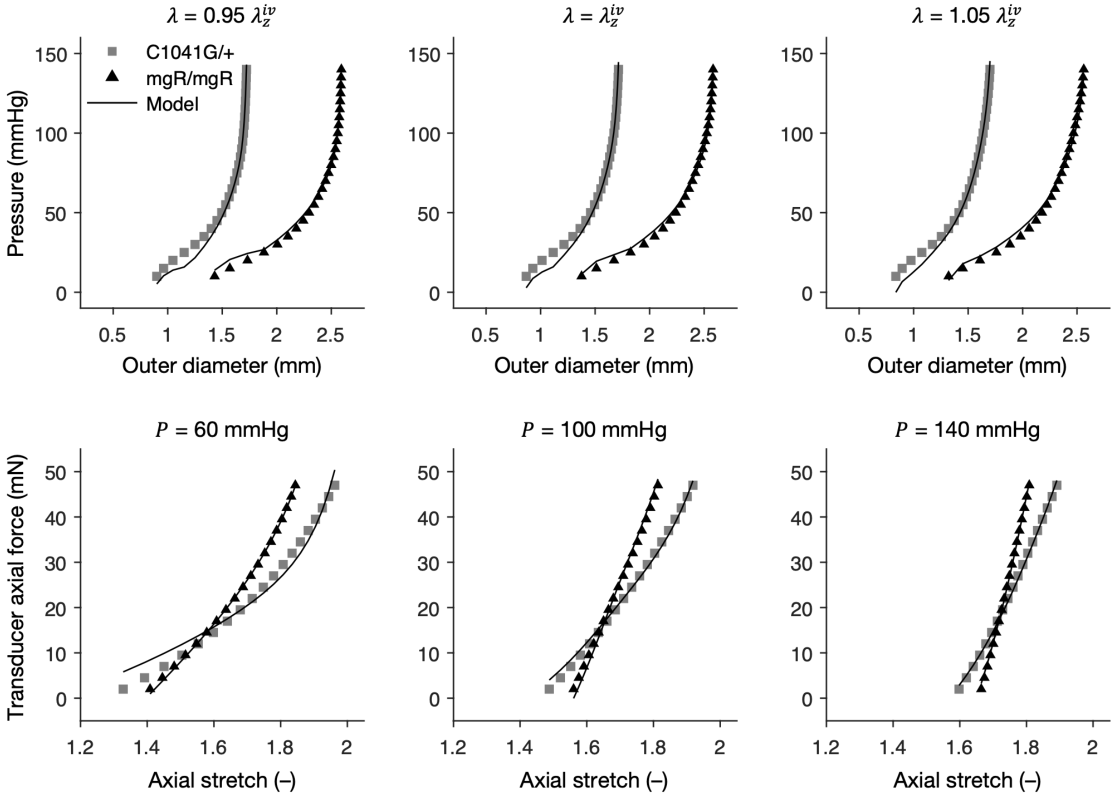

Figure B.1: Material characterization of the Marfan ascending aorta using synthetic pressure-diameter and force-stretch data (symbols) generated from bulk material parameters from mild $Fbn1^{C1041G/+}$ and severe $Fbn1^{mgR/mgR}$ mice [1]. All protocols were fit using one bilayered model (lines) with G&R between states represented by the two groups. Elastin mass and deposition stretches are held constant between the two states, while smooth muscle and collagen mass, stored energy parameters, and deposition stretches are allowed to evolve.

Appendix C Collagen cross-linking vs. deposition stretch

The constitutive relation for the stored energy contribution of collagen-dominated matrix in the aortic wall is given by

$$\hat{W}^c = \frac{c_1^c}{4c_2^c}\Big(\exp\big(c_2^c(I_4^c-1)^2\big)-1\Big), \tag{1}$$

where $\{c_1^c, c_2^c\}$ are material parameters for collagen, and $\{I_4^c\}$ is the coordinate invariant measure of the collagen deformation. While both parameters can model changes in collagen properties, c_1^c has a linear scaling effect, and G^c has a nonlinear effect (Figure C.1).

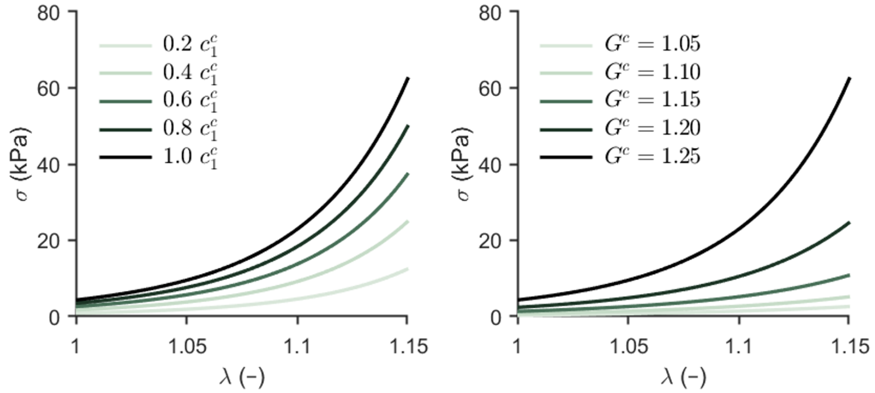

Figure C.1: Behavior of the Fung exponential constitutive relation for collagen (Equation 2.4). (a) Parameter sweep for c_1^c from 20%-–100% baseline value (Table 1), analogous to altered collagen cross-linking. (b) Parameter sweep for G^c from 1.05–1.25, modeling collagen deposition stretch. Baseline values: c_1^c = 665.6 kPa, G^c = 1.25.

Appendix D Mechanobiological insult parameters

Figure D.1 depicts examples of how the combined insult model was parameterized, using the experimentally measured elastin porosity and prescribed insult parameters as inputs.

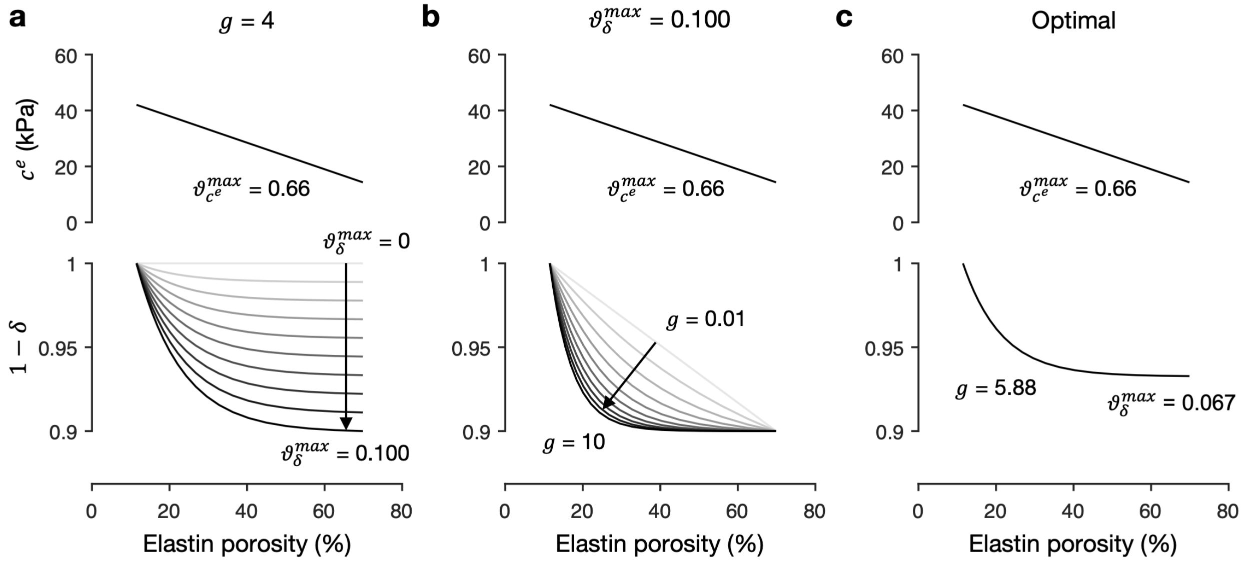

Figure D.1: Pseudo-time evolutions for diminished elastic fiber integrity and dysfunctional mechanosensing as functions of elastin porosity (Equations 2.5–2.6). Shown are values for elastin modulus c^e and cellular mechanosensing $1-\delta$. (a) Parameter sweeps for maximal mechanosensing compromise ($\vartheta_\delta^{max} \in [0, 0.100]$), with fixed progression rate g = 4. (b) Parameter sweep for progression rate $g \in [0.01, 10]$ with fixed maximal mechanosensing compromise ($\vartheta_\delta^{max} = 0.100$). (c) Representative optimal insult parameters, reported in Table 2. Mechanoregulation insults (reduced collagen deposition stretch G^c) were treated similarly. In all cases, experimentally measured elastin porosity increases from 11.5% to 69.9% [1]. At each point, the insult profile varies axially as shown in Figure 4.

Appendix E Individual insults

Simulations were run with each insult contributor individually applied: reduced elastic fiber integrity (Figure E.1) and dysfunctional cellular mechanosensing (Figure E.2) and mechanoregulation (Figure E.3), along with altered smooth muscle-to-collagen turnover ratio η and shear-to-intramural gain ratio K_{τ_w}/K_σ. Rather than elastin porosity, the results are plotted against experimentally measured inner diameter normalized to the average wild-type value. Note here that model results were not fitted to the experimental data, determined instead from parameter sweeps for $\vartheta_{c^e}^{max}$, ϑ_{δ}^{max}, and $\vartheta_{G^c}^{max}$.

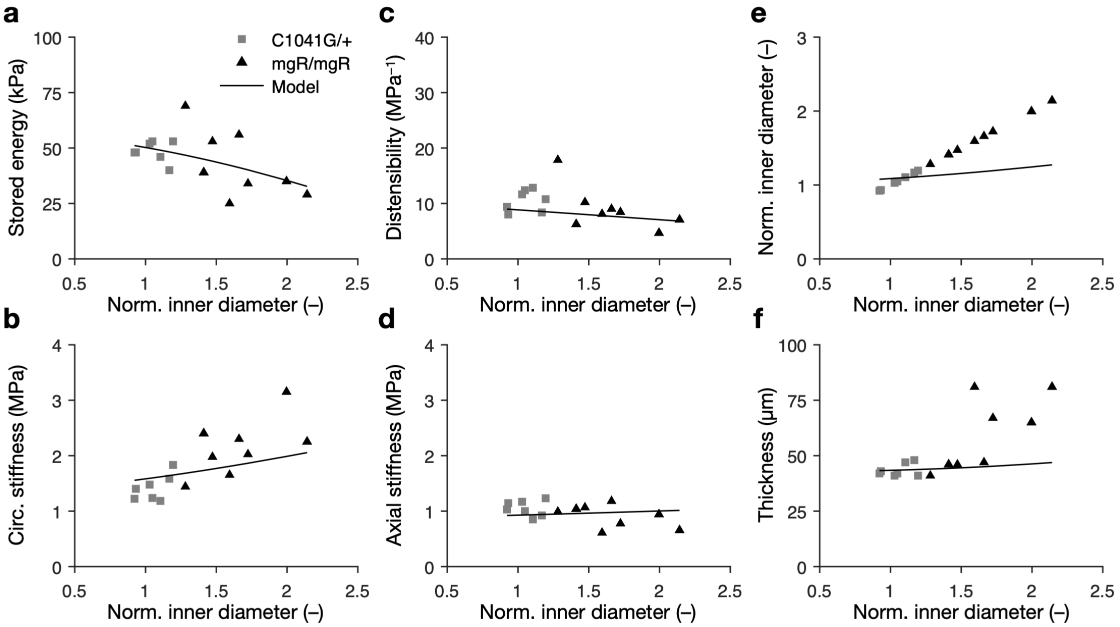

Figure E.1: Simulating diminished elastic fiber integrity alone (0–66% based on histological data). Experimental data (symbols) from $Fbn1^{C1041G/+}$ (squares) and $Fbn1^{mgR/mgR}$ (triangles) aortas and model results (lines) for vessel geometry and mechanical properties at diastolic pressure (80 mmHg) plotted against normalized inner diameter (experimentally measured ratio of inner diameter relative to wild-type). The model captured a decrease in stored energy consistent with experimental observations, but only modest increases in circumferential stiffness, normalized inner diameter, and thickness, thus underestimating most of the experimental data.

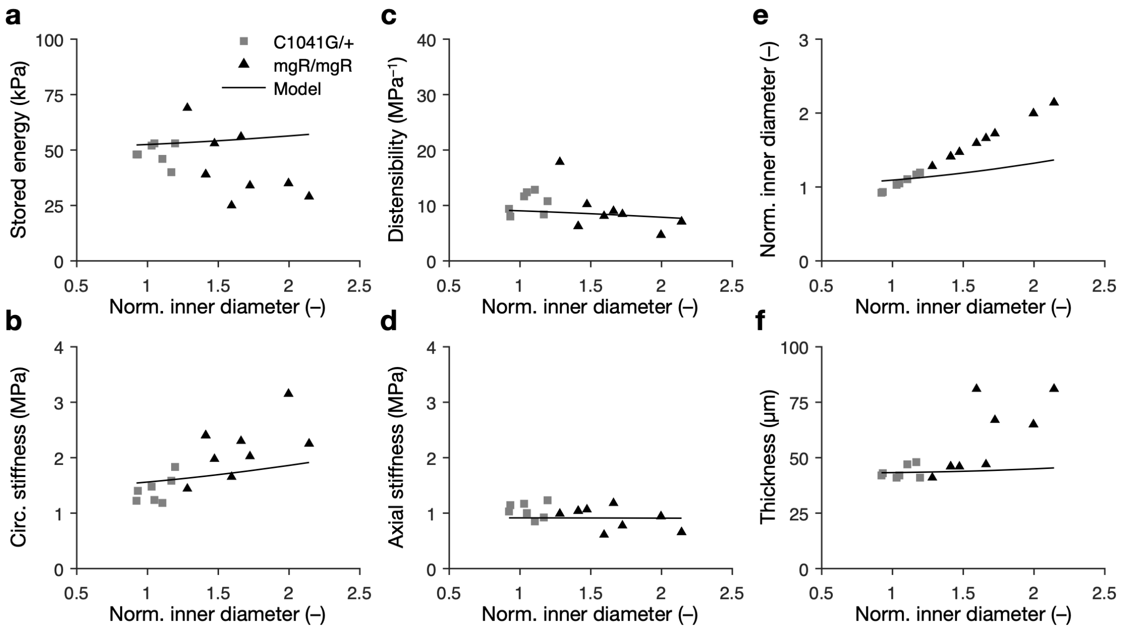

Figure E.2: Same as Figure E.1 but simulating dysfunctional mechanosensing alone ($\delta = 0$–0.150, progressing linearly) with no changes to elastic fiber integrity. In contrast to effects of diminished elastic fiber integrity (Figure E.1), dysfunctional mechanosensing associated with increased stored energy, contrary to trends in the experimental data. This simulation resulted in more dilatation compared to diminished elastic fiber integrity alone but continued to underestimate geometric quantities.

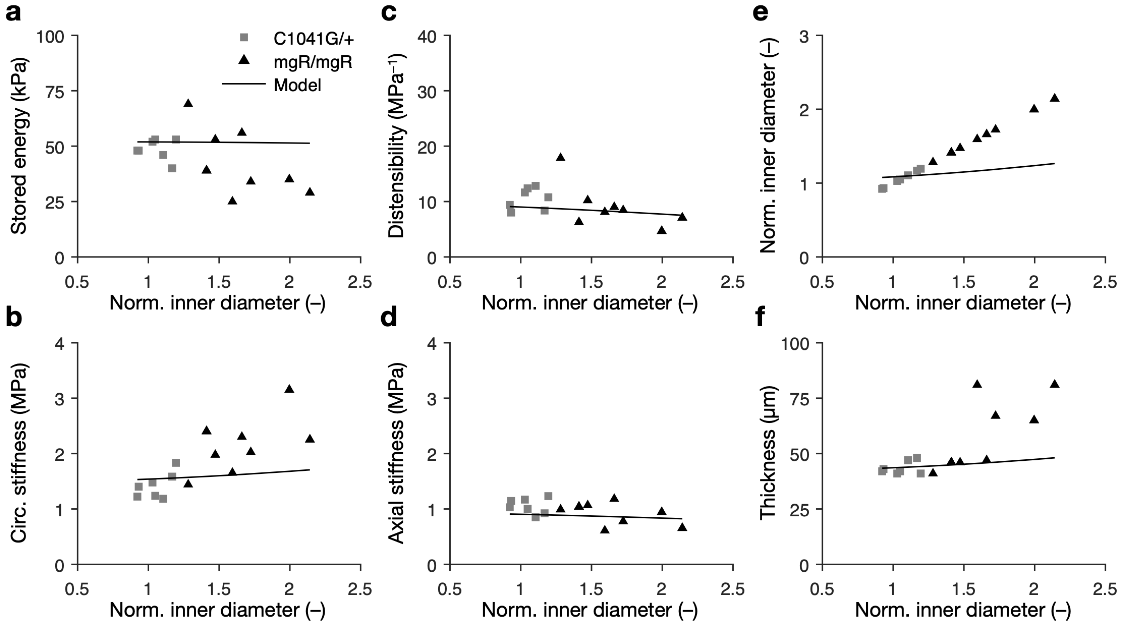

Figure E.3: Same as Figure E.1 but simulating dysfunctional mechanoregulation alone (0–1.2%, progressing linearly). Effects on stored energy were relatively insensitive to mechanoregulation insults, and experimental quantities were mostly underestimated.

Appendix F Sensitivity to progression rate

An illustrative parameter sweep was performed in which values for ϑ_{c^e}, ϑ_δ, and ϑ_{G^c} were fixed and g was varied from 1–10 to assess the sensitivity of model results to the progression rate parameter. Although the predicted trends for stored energy were relatively insensitive to variations in g, lower values of g associated with linear increases in circumferential stiffness and nonlinear increases in normalized inner diameter and thickness (Figure F.1).

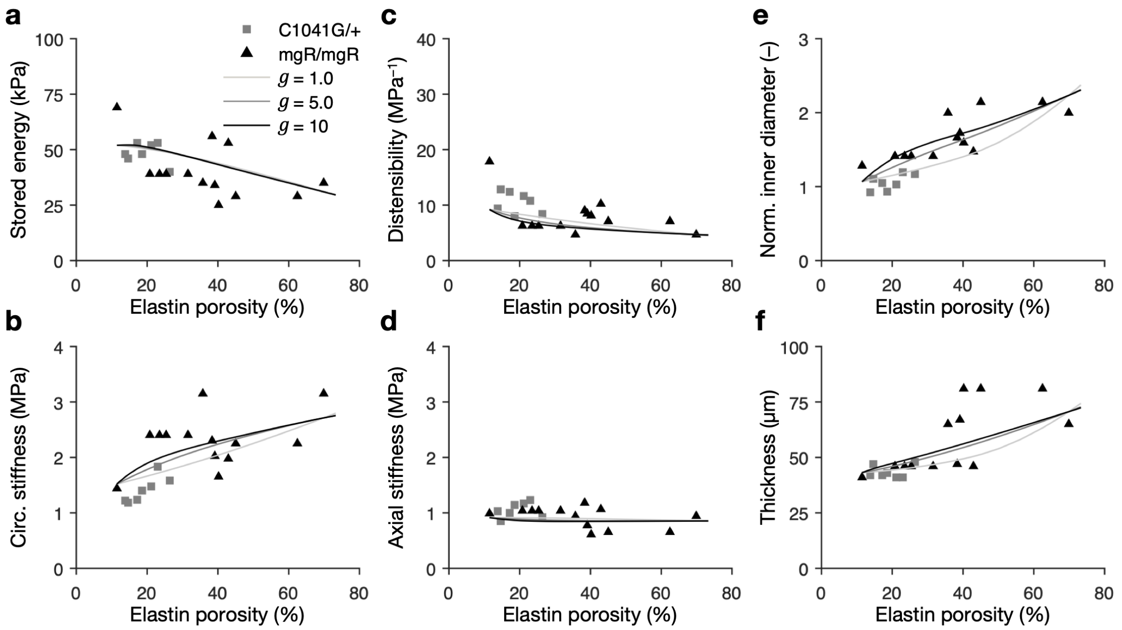

Figure F.1: Experimental data and combined insult model results for vessel geometry and mechanical properties at diastolic pressure (80 mmHg) plotted against elastin porosity. The elastic fiber integrity insult and optimal values for mechanosensing and mechanoregulation insults were fixed ($\vartheta_{c^e} = 0.660$, $\vartheta_\delta = 0.0672$, $\vartheta_{G^c} = 0.0072$) while the progression rate g was varied between the upper and lower bounds.

Appendix G Optimal combined insult parameters

Plotting optimal parameters with respect to one another revealed strong correlations (Figure G.1), particularly between maximal contributions of dysfunctional mechanosensing and mechanoregulation, though less significant for progression rate. That simulations were similar for dysfunctional mechanosensing (assessing matrix) and mechanoregulation (assembling matrix) was not unexpected, since both of these mechanobiological functions depend on both integrins and actomyosin activity. Recall that altered integrin signaling and decreased contractile protein expression have been observed in both Marfan mouse models.

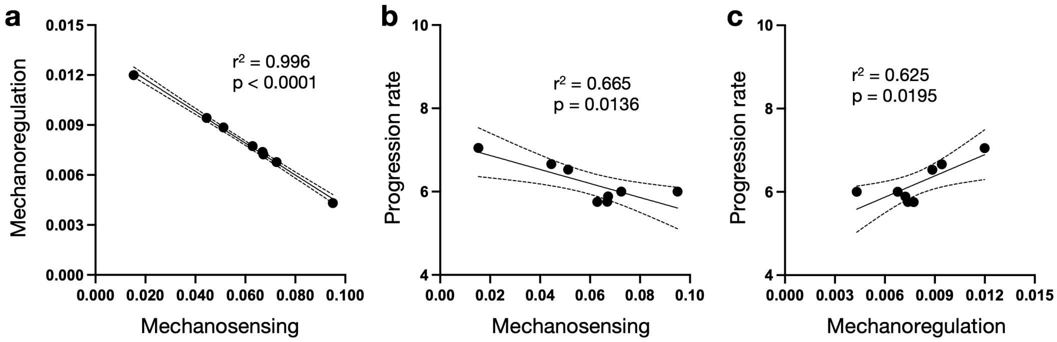

Figure G.1: Correlations between optimal insult parameters for mechanosensing $\vartheta_{\delta}^{max*}$, mechanoregulation $\vartheta_{G^c}^{max*}$, and progression rate g^* from several optimization trials, with 95% confidence intervals indicated by the dashed lines: (a) mechanoregulation and mechanosensing, (b) progression rate and mechanosensing, (c) progression rate and mechanoregulation.

References

[1] Cavinato C, Chen M, Weiss D, Ruiz-Rodríguez MJ, Schwartz MA, Humphrey JD. 2021 Progressive microstructural deterioration dictates evolving biomechanical dysfunction in the Marfan aorta. *Frontiers in Cardiovascular Medicine* **8**, 800730.

[2] Bellini C, Bersi M, Caulk A, Ferruzzi J, Milewicz D, Ramirez F, Rifkin D, Tellides G, Yanagisawa H, Humphrey J. 2017 Comparison of 10 murine models reveals a distinct biomechanical phenotype in thoracic aortic aneurysms. *Journal of The Royal Society Interface* **14**, 20161036.

[3] Ferruzzi J, Madziva D, Caulk A, Tellides G, Humphrey J. 2018 Compromised mechanical homeostasis in arterial aging and associated cardiovascular consequences. *Biomechanics and Modeling in Mechanobiology* **17**, 1281–1295.

[4] Latorre M, Humphrey JD. 2018 Modeling mechano-driven and immuno-mediated aortic maladaptation in hypertension. *Biomechanics and Modeling in Mechanobiology* **17**, 1497–1511.
